# Lake Keuka Medical and Surgical Association

**Published:** 1903-09

**Authors:** W. W. Smith

**Affiliations:** Secretary, Avoca, N. Y.


### SOCIETY PROCEEDINGS.

### Lake Keuka Medical and Surgical Association.

REPORTRD BY W. W. SMITH, M. D., Secretary, Avoca, N. Y.

The fourth annual meeting of the Lake Keuka Medical and Surgical Association was held at Grove Springs, August 11 and 12, 1903.

The president, DR. ARTHUR W. BOOTH, of Elmira, took the chair and called the meeting to order at 12 o'clock noon, Tuesday, August 11, after which the minutes of the meeting of 1902 were read and approved.

The treasurer's report showing a balance of $14.00 in the treasury after paying all bills of 1902, was presented and approved.

The president then presented his annual address entitled, Surgical intervention in typhoid fever perforations.

A recess for dinner was taken and the association convened at 2 o'clock p.m.

The president's address was discussed by Henry R. Hopkins, of Buffalo; H. P. Jack, of Canisteo; H. D. Wey, of Elmira; C. L. Stiles, of Owego; Ross G. Loop, of Elmira, and John D. Miller, of Corning.

A paper was read by William Warren Potter, of Buffalo, entitled, Some diseases of the respiratory tract from a pension examiner's viewpoint.

The discussion was opened by C. L. Stiles, of Owego, who was followed by Warren L. Babcock and I. P. Smith, of Bath; Geo. M. Case, of Elmira; R. P. Bush, of Horseheads, and closed by the author.

The president appointed an executive committee, as follows: C. S. Parkhill, Steuben; Henry Flood, Chemung; M. L. Bennett, Schuyler; C. E. Doubleday, Yates; S. R. Wheeler, Ontario; C. L. Stiles, Tioga, and F. R. Driesbach, of Livingston.

A paper then was read by Wm. B. Jones, of Rochester, entitled, Prostatic hypertrophy and similar conditions.

The discussion was opened by Warren L. Babcock, of Bath, and closed by the reader.

The next paper was read by Ernest Wende, of Buffalo, on Ointments and pastes.

A paper entitled, Blood examination as an aid to diagnosis, was read by Clayton R. Haskell, of the Soldiers' Home, Bath, which was discussed by Ross G. Loop, of Elmira, and C. L. Stiles of Owego.

A paper was then read by H. D. Wey, of Elmira, subject, Pancreatitis. It was discussed by C. W. M. Brown, of Elmira; Lewis W. Rose, of Rochester, and closed by the author.

Henry R. Hopkins, of Buffalo, then read a paper entitled, Malignant endocarditis, which was discussed by H. R. Ainsworth, of Addison; C. W. M. Brown, of Elmira, and the discussion was closed by the author. A practical demonstration of making a diagnosis of prolapsus of the kidney, was the title of a dissertation by Augustin H. Goelet, of New York. The paper was discussed by H. P. Jack, of Canisteo, and closed by Dr. Goelet.

A motion was made by Dr. Wey and seconded by Dr. Jacobs that the question of changing the date of meeting from August to July be referred to the executive committee.

A recess was taken at 6 o'clock until Wednesday, August 12, 9.15 a.m.

The first paper read at the morning session was entitled, Perforative appendicitis, by A. L. Beahan, of Canandaigua, which was discussed by John D. Miller, of Corning, and the author.

The next paper was read by John O. Roe, of Rochester, entitled, Diseased tonsil considered as a seat of infection, the causation of rheumatism and allied diseases. Paper discussed by R. P. Bush, of Horseheads; G. M. Case, Elmira; Sargent F. Snow, Syracuse; P. K. Stoddard, Prattsburg; William Warren Potter, of Buffalo, and closed by the reader.

A paper was read by Sargent F. Snow, of Syracuse, entitled, Free drainage and ice in the early stage of mastoiditis. Discussed by Henry Flood and Geo. M. Case, of Elmira; John O. Roe, of Rochester, and closed by the author.

A paper was read by C. S. Greenleaf, of Rochester, entitled, Therapeutics of the Röntgen ray, which was discussed by Frank W. Ross, of Elmira; John O. Roe, of Rochester; P. K. Stoddard, of Prattsburg, and in closing by the reader.

The next paper read was by Geo. M. Case, of Elmira, entitled, Ophthalmia neonatorum, and was discussed by R. P. Bush, of Horseheads.

The executive committee reported the following: Resolved, that the time of meeting of the Lake Keuka Medical and Surgical Association hereafter shall be on the Thursday and Friday occurring nearest the first full moon of the month of July. Carried.

The committee reported the following nominations for officers for the next year: president, A. L. Beahan, of Ontario; vice-president, P. L. Alden, of Steuben; secretary and treasurer, W. W. Smith, of Steuben. The nominees reported by the committee were elected. A committee of which Dr. Stiles was chairman was appointed to confer with Dr. W. W. Potter in regard to printing the papers of the meeting in the BUFFALO MEDICAL JOURNAL. Attendance, 75. Adjourned sine die.

